# Peer Relatedness, School Satisfaction, and Life Satisfaction in Early Adolescence: A Non-recursive Model

**DOI:** 10.3389/fpsyg.2021.641714

**Published:** 2021-03-09

**Authors:** René Gempp, Mònica González-Carrasco

**Affiliations:** ^1^Facultad de Economía y Empresa, Universidad Diego Portales, Santiago, Chile; ^2^Research Institute on Quality of Life (IRQV), Universitat de Girona, Girona, Spain

**Keywords:** peer relatedness, school belonging, school satisfaction, life satisfaction, early adolescence, non-recursive model, reciprocal effects

## Abstract

Cumulative evidence suggests that, for children and adolescents, peer relatedness is an essential component of their overall sense of belonging, and correlates with subjective well-being and school-based well-being. However, it remains unclear what the underlying mechanism explaining these relationships is. Therefore, this study examines whether there is a reciprocal effect between school satisfaction and overall life satisfaction (Hypothesis 1), and whether the effect of peer relatedness on life satisfaction is mediated by school satisfaction (Hypothesis 2). A non-recursive model with instrumental variables was tested with econometric and structural equation modeling methodologies, using a cross-sectional sample of *n* = 5,619 Chilean early adolescents (49.2% girls), aged 10, 11, and 12 (46.13, 44.99, and 8.88% respectively). Results were highly consistent across methods and supported the hypotheses. First, the findings confirmed a significant reciprocal influence between school satisfaction and overall life satisfaction, with a greater impact from school to life satisfaction. Second, the effect of peer relatedness on overall life satisfaction was fully mediated by school satisfaction. The study further suggests the importance of considering reciprocal effects among domain-specific satisfaction and overall life satisfaction and illustrates the application of non-recursive models for this purpose.

## Introduction

Around the same time that Baumeister and Leary (1995) introduced their *Belongingness Hypothesis* (for a review, see Oyanedel and Páez, 2021, in this special issue), Goodenow and Grady (1993, p. 80) conceptualized school belonging as “*the extent to which students feel personally accepted, respected, included, and supported by others in the school social environment*.” Further theoretical development and empirical research showed that a strong sense of belonging to the school predicts positive academic outcomes and contributes to children and adolescents' mental health and well-being (for reviews, see Fredricks et al., 2004; Martin and Dowson, 2009; Slaten et al., 2016; Allen and Kern, 2017).

Both the original Belongingness Hypothesis (Baumeister and Leary, 1995; Baumeister, 2012) and the foundational definition of school belonging (Goodenow and Grady, 1993) emphasize the role of “others” as sources of a sense of belonging. In school setting those others can be peers, teachers, or anyone in the school environment (Slaten et al., 2016). Indeed, in well-known measures of school belonging such as the Psychological Sense of School Membership scale (PSSM; Goodenow, 1993; Ye and Wallace, 2014), the Hemingway Measure of Adolescent Connectedness (HMAC; Karcher, Unpublished) or the School Connectedness Scale (SCS; Lohmeier and Lee, 2011), students are asked about their perceived connectedness with three targets: peers, teaches, and school.

Given that classmates are an essential component of school experience (Huebner et al., 2014), peer relatedness is a key ingredient of school belonging (Kingery et al., 2011; Allen and Kern, 2017; Mikami et al., 2017). Peer relatedness can be broadly conceptualized as the feeling that results from the sum of positive peer interactions with classmates (Schmidt et al., 2019, 2020), and the subjective experience of getting along with peers, being accepted, supported and valued by them (Furrer and Skinner, 2003).

Cumulative evidence reveals that an important outcome associated with peer relatedness at school is subjective well-being (Gross-Manos, 2014; Slee and Skrzypiec, 2016). These findings are particularly relevant due to the growing interest in the study of subjective well-being in childhood and adolescence (for a review, see Ben-Arieh et al., 2014), driven by the need for indicators to aid decision making within the context of public policies aimed at this population (Casas, 2011; Oyanedel et al., 2015).

Subjective well-being is usually conceptualized as how people evaluate their own lives, both in general and for specific life domains. It is a multidimensional construct, composed of a cognitive process (life satisfaction) and two affective processes, namely positive and negative affect (Diener et al., 1999).

Several studies have examined the relationship between peer relatedness and school well-being's cognitive component, i.e., school satisfaction (Oriol et al., 2017). For instance, Jiang et al. (2013) examined the effect of three sources of school-related social support on school satisfaction and found that peer support significantly explained school satisfaction. In the same vein, Muscarà et al. (2018) found that peer support in middle school predicts school satisfaction. On the other hand, Tian and colleagues (Tian et al., 2015, 2016a,b) conceptualize and measure school-related subjective well-being as being comprised of school satisfaction, and affective experience (positive and negative), and present compelling evidence on the association between peer relatedness and school-related subjective well-being, in children and adolescents.

Other studies have examined the association between peer relatedness and global subjective well-being, focusing on cognitive or affective processes. For example, Schwarz et al. (2012) reported that peer acceptance predicts life satisfaction in adolescents from 11 cultures, and that the effect was moderated by the importance of family values in each culture. On the other hand, in a two-wave panel study with Filipino adolescents, King (2015) found that peer relatedness predicts positive and negative affect. And two recent studies using intensive longitudinal designs (Schmidt et al., 2019, 2020) shown that peer relatedness at school predicts positive and negative affect in German children.

### Potential Reciprocal Effect Between School Satisfaction and Life Satisfaction

An unresolved question is clarifying the underlying mechanism by which peer relatedness is associated with subjective well-being and the role that school-based well-being plays in this relationship.

On the one hand, self-determination theory (Deci and Ryan, 2012) and Cognitive-experiential self-theory (Epstein, 1998) are usually cited for justifying a direct effect of peer relatedness on subjective well-being. Most studies in the field assume, explicitly or implicitly, a proximal effect according to which feeling accepted by peers satisfies the basic human need for belonging and that the relief of this need increases subjective well-being. Other studies assume a distal effect, in which peer relatedness promotes healthy psychological development, boosting overall positive affect and life satisfaction (e.g., Schmidt et al., 2019).

On the other hand, because social interaction with peers is a crucial component of school experience, it is logical to conclude that peer relatedness directly influences children's school satisfaction, as suggested by the previously summarized studies. Moreover, recent work in social identity theory applied to school social environment (Turner et al., 2014; Reynolds et al., 2017a,b; Simonsen and Rundmo, 2020) provides a framework to endorse a direct effect of peer relatedness on school satisfaction^1^.

**Figure 1:**
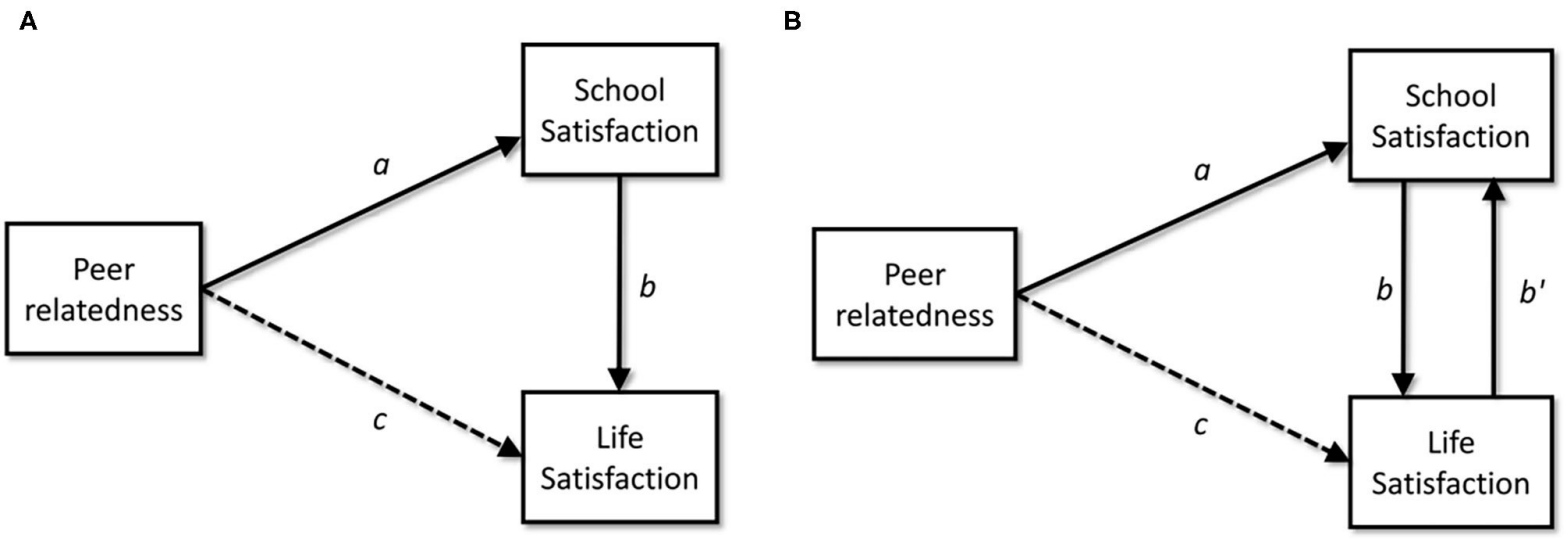
**(A)** Basic mediational model. **(B)** Mediational model with reciprocal effects between school satisfaction and life satisfaction.

A way to reconcile these findings is to assume that the effect of peer relatedness on life satisfaction is mediated by school satisfaction. As shown in Figure 1A, peer relatedness at school would have a direct effect on school satisfaction and an indirect effect on life satisfaction. If this assumption is correct, paths *a* and *b* will be significant, and path *c* non-significant. A few studies (Suldo et al., 2008; Danielsen et al., 2009) have addressed this hypothesis, but their results indicate only a partial mediation effect (path *c* significant) rather than a full mediation (path *c* non-significant).

A potential flaw of this basic mediational model is that it assumes a unidirectional effect between school satisfaction and life satisfaction. Nevertheless, since Diener (1984) introduced the distinction between bottom-up and top-down models, cumulative evidence suggests that, for some life domains, the relationship between domain-specific satisfaction and overall life satisfaction could be bidirectional. For instance, reciprocal effects have been reported between life satisfaction and marriage satisfaction (Headey et al., 2005), team satisfaction (Chen et al., 2018), or job satisfaction (Bowling et al., 2010; Bialowolski and Weziak-Bialowolska, 2020), among others.

Although to the best of our knowledge, no published studies have examined the directionality of the relationship between school satisfaction and life satisfaction in early adolescents, we propose that a reciprocal causal link should be seriously considered. Given the evidence of directionality between domain satisfaction and overall life satisfaction in various life domains, there is no reason to presume a priori that school satisfaction should be an exception.

Take, for instance, the well-documented reciprocal relationship between job satisfaction and life satisfaction (Bowling et al., 2010; Bialowolski and Weziak-Bialowolska, 2020). Early adolescents' school experience bears certain similarities to adults' work experience: it is a place where they spend many hours a week, and both are a source of social relationships and a sense of belonging, in addition to satisfying needs for relatedness, autonomy, and competence. Just as life satisfaction partially influences job satisfaction judgments (Bowling et al., 2010), it is plausible to assume that overall life satisfaction affects early adolescents' evaluation of school satisfaction. Just as job satisfaction directly affects life satisfaction, school satisfaction can be considered a determinant of early adolescents' life satisfaction. It is reasonable for both specific domains to expect a spillover effect with life satisfaction and job or school satisfaction mutually affecting each other.

The proposed reciprocal effect between school satisfaction and overall life satisfaction is presented in Figure 1B. Drawing on the widespread evidence on school relevance in early adolescents' lives, we specifically hypothesize (H1) that the effect of school satisfaction on life satisfaction will be greater than the effect of life satisfaction on school satisfaction. In Figure 1B, our first hypothesis proposes that path *b* > path *b*′.

Regarding the role of peer relatedness, based on previous research, we hypothesize (H2) that the relationship between peer relatedness at school and overall life satisfaction in early adolescence is fully mediated by overall school satisfaction. If this hypothesis is correct, in Figure 1B paths *a* and *b* will be significant, and path *c* will not.

### Study Aim and Hypotheses

In summary, this study aims to test two hypotheses concerning the non-recursive mediational model shown in Figure 1B. Our first hypothesis (H1) proposes a significant bidirectional relationship between children's school and life satisfaction, but that the effect from school to life satisfaction is greater than the opposite direction (path *b* > path *b*′). The second hypothesis (H2) is that the effect of peer relatedness on life satisfaction is fully mediated by school satisfaction (paths *a* and *b* as significant; path *c* as non-significant).

The best approach to test bidirectional relationships is to use panel data. When this is not possible, a tenable alternative is to use non-recursive structural equation models and rely on so-called instrumental variables (Paxton et al., 2011), as will be explained in the Method section below.

## Method

### Participants

We used data from the third wave of the Chilean Early-Childhood Longitudinal Survey (ELPI, 2017–2018), a nationally representative study conducted by the Chilean Ministry of Education to better understand sociodemographic characteristics and developmental outcomes of Chilean children and their families. The ELPI consisted of two household information-gathering visits at home, in which surveys were applied for children ages seven or more, and their caregivers. Also, psychological instruments were applied to assess cognitive, socioemotional, and physical aspects. Trained research assistants from undergraduate psychology programs, collected the data. Informed consent was obtained from all individual participants. The ELPI study was evaluated and approved by several ethical committees (see *Ethic Statements*, for details).

The third wave of the ELPI surveyed 17,307 boys and girls born between January 1, 2006, and December 31, 2016. Only the survey booklet for participants ages ten or more (thus, belong to the category of early adolescence; see Lansford and Banati, 2018) included questions about school and life satisfaction. Consequently, the current study draws on the 5,619 early adolescents (49.2% girls) aged 10, 11, and 12 (46.13, 44.99, 8.88%, respectively) who were asked about their life and school satisfaction. Participants were enrolled in public (48.83%), voucher private (46.98%), and non-subsidized schools (4.19%), in 2nd (0.43%), 3rd (3.17%), 4th (20.14%), 5th (41.82%), 6th (27.27%), 7th (7.12%), and 8th (0.05%) grade. Note that primary school in Chile is compulsory and divided into eight grades, for students aged 6–14.

### Measures

An attractive feature of the ELPI is that it collects information from the children's perspective. The survey includes a single-item scale on overall life satisfaction that asks, “*to what extent are you satisfied with your life as a whole?*” It also includes a question to evaluate overall satisfaction with school life: “*to what extent are you satisfied with your school experience?*” Both questions use a 1–7 scale, anchored from “not satisfied at all” to “very satisfied.”

The questionnaire does not incorporate a peer relatedness scale, but it does embody several questions that can be used to assess it. Grounded in the conceptual definition of peer relatedness and from the review of other measures (e.g., Mikami et al., 2017; Devine et al., 2018; Schmidt et al., 2019), we select five questions to develop a scale: “*How is your relationship with your classmates*?” (very bad =1, bad = 2, more or less = 3, good = 4, very good = 5); “*My classmates think I have good ideas*” (yes =1, no = 0); “*I feel lonely in the class*” (never = 4, sometimes = 3, almost always = 2, always = 1); “*I have a good time with my classmates*” (never = 1, sometimes = 2, almost always = 3, always = 4); “*I have a hard time in the classroom*” (never = 4, sometimes = 3, almost always = 2, always = 1). To extract the maximum information from the items, and because of the mixed format of the answers options, we created a unidimensional scale using Item Response Theory instead of Classical Test Theory.

We calibrated the items using Samejima Graded Response Model 1997, with the MHRM estimation algorithm, through the IRTPRO 2.1 software (Cai et al., 2011). The scale fit was excellent (RMSEA = 0.01), with factor loadings from 0.61 to 0.84 and construct reliability ρ = 0.86. Item parameters are presented as Supplementary Material. We computed a scale score from response patterns, using *Expected a Posteriori* (EAP) method, as is usual in IRT applications (Brown and Croudace, 2015), with mean = 0 and standard deviation = 1.

### Analytical Strategy: Instrumental Variables

Non-recursive models cannot be estimated unless we use Instrumental Variables (IV). In short, IVs are methodological devices used to resolve problems of endogeneity, which occur when an explanatory variable correlates with the error term of the dependent variable. If school satisfaction and life satisfaction have reciprocal effects, both correlate with the other one's error term; in fact, all non-recursive models suffer from endogeneity problems and need IVs. In an equation system as IV → X → Y, a IV is any variable that (a) has a strong and significant direct effect on X but (b) is not correlated with the disturbance term of Y. The first assumption (*instrument strength*) is necessary to achieve efficient estimates. The second assumption is called *instrument validity* and ensures consistent estimates. Both assumptions can be statistically tested. For instance, in the model presented in Figure 1B, an IV for school satisfaction could be any variable that affects school satisfaction but does not correlate with the disturbance term of life satisfaction. An introduction to endogeneity and IVs can be found in any intermediate level econometric text. The monograph of Paxton et al. (2011) offers a thoughtful presentation regarding non-recursive models.

The model shown in Figure 1B requires at least one IV for school satisfaction and another one for life satisfaction to be identified. Due to technical details beyond this paper's scope (see Paxton et al., 2011 for an explanation) it is advisable to estimate an over identified model, which requires at least two IVs for each dependent variable.

For school satisfaction, we select as our first IV the question “*How much do you like physical education classes?”* (I like them very much = 5, I like them = 4, I neither like nor dislike them = 3, I dislike them = 2, I dislike them a lot =1), because of the known effects of physical education classes on school satisfaction (Garn and Cothran, 2006; Dismore and Bailey, 2011) but the lack of documented effects on life satisfaction. As the second IV, we used the scale of attentional problems of Child Behavior Checklist (Achenbach and Ruffle, 2000; CBCL-2), applied to the children surveyed in the third wave of the ELPI, and validated by ELPI's technical staff. The underlying rationale is that early adolescents' with attentional problems have a greater probability of experiencing school adjustment difficulties and, consequently, lower school satisfaction (Ogg et al., 2016). However, attentional problems do not necessarily affect life satisfaction.

For life satisfaction, we selected as our IVs the questions “*In the last week, did you and someone in your family play together at home?*” (yes = 1, no = 0) and “*Does [name of primary caregiver] know where you are after school?*” (does not know = 0, has some idea = 0, knows a lot = 1). We chose these IVs because home-related positive experiences and parental knowledge about early adolescents' everyday life have an established effect on their life satisfaction (Padilla-Walker et al., 2008). Still, there are no logical reasons to presume that these specific home-related experiences must affect school satisfaction.

We assess the strength and validity of our putative IVs using statistical tools developed in econometrics (Wooldridge, 2002; Paxton et al., 2011). The results, presented as Supplementary Material, show that all our IVs are strong and valid.

Following Paxton et al. (2011) recommendation, we use two approaches to estimate the hypothesized model, and we check the results' consistency across methods as evidence of robustness. All analyses were conducted using Stata 16.

First, we use standard econometric methods for IVs, namely Two-Stage Least Squares (2SLS) and Three-Stage Least Squares (3SLS). In addition to providing statistical tests for testing the IVs' strength and validity, econometric methods are more robust to structural misspecifications than system-wide estimators used in Structural Equation Modeling (SEM) approach, but at the cost of not providing model fit indices.

We also estimate the model with maximum likelihood SEM, with standard errors and Chi-square adjusted with the Satorra and Bentler (1994) correction. Compared with 2SLS and 3SLS, SEM methods allow evaluating the fit and stability of the model, besides facilitating the estimation of indirect effects needed to test mediational hypotheses.

## Results

Table 1 shows descriptive statistics for all the variables used in the analysis. As expected, life satisfaction and school satisfaction are associated (*r* = 0.41, *p* < 0.001), and the correlation between peer relatedness and school satisfaction (*r* = 0.36, *p* < 0.001) is greater than its correlation with life satisfaction (*r* = 0.26, *p* < 0.001). The IVs correlate higher with their respective instrumented variables than with the other outcome.

**Table 1:** Means, standard deviations, and correlations of the variables used in the model.

**Variable**	**Mean**	**SD**	**Min**	**Max**	**1**	**2**	**3**	**4**	**5**	**6**
1. Life satisfaction	6.49	1.07	1	7						
2. School satisfaction	6.17	1.20	1	7	0.41^***^					
3. Peer relatedness^a^	0.00	1.00	−3	3	0.26^***^	0.36^***^				
4. Liking physical education	4.53	0.74	1	5	0.11^***^	0.16^***^	0.20^***^			
5. Attentional problems	3.81	3.40	0	20	−0.11^***^	−0.16^***^	−0.14^***^	−0.04^**^		
6. Playing with family	0.69	0.46	0	1	0.11^***^	0.08^***^	0.12^***^	0.10^***^	−0.04^**^	
7. Parental knowledge	0.89	0.31	0	1	0.13^***^	0.08^***^	0.13^***^	0.02	−0.03^*^	0.06^***^

* *p < 0.05*,

** *p < 0.01*,

*** *p < 0.001*.

a *Expected a posteriori (EAP) score; mean and standard deviation were scaled to 0 and 1, respectively, during the estimation process*.

Before estimating the model, we standardized (mean = 0, standard deviation = 1) all variables, except the dichotomous ones, which we kept with the 0/1 format to facilitate the results' interpretation.

**Figure 2:**
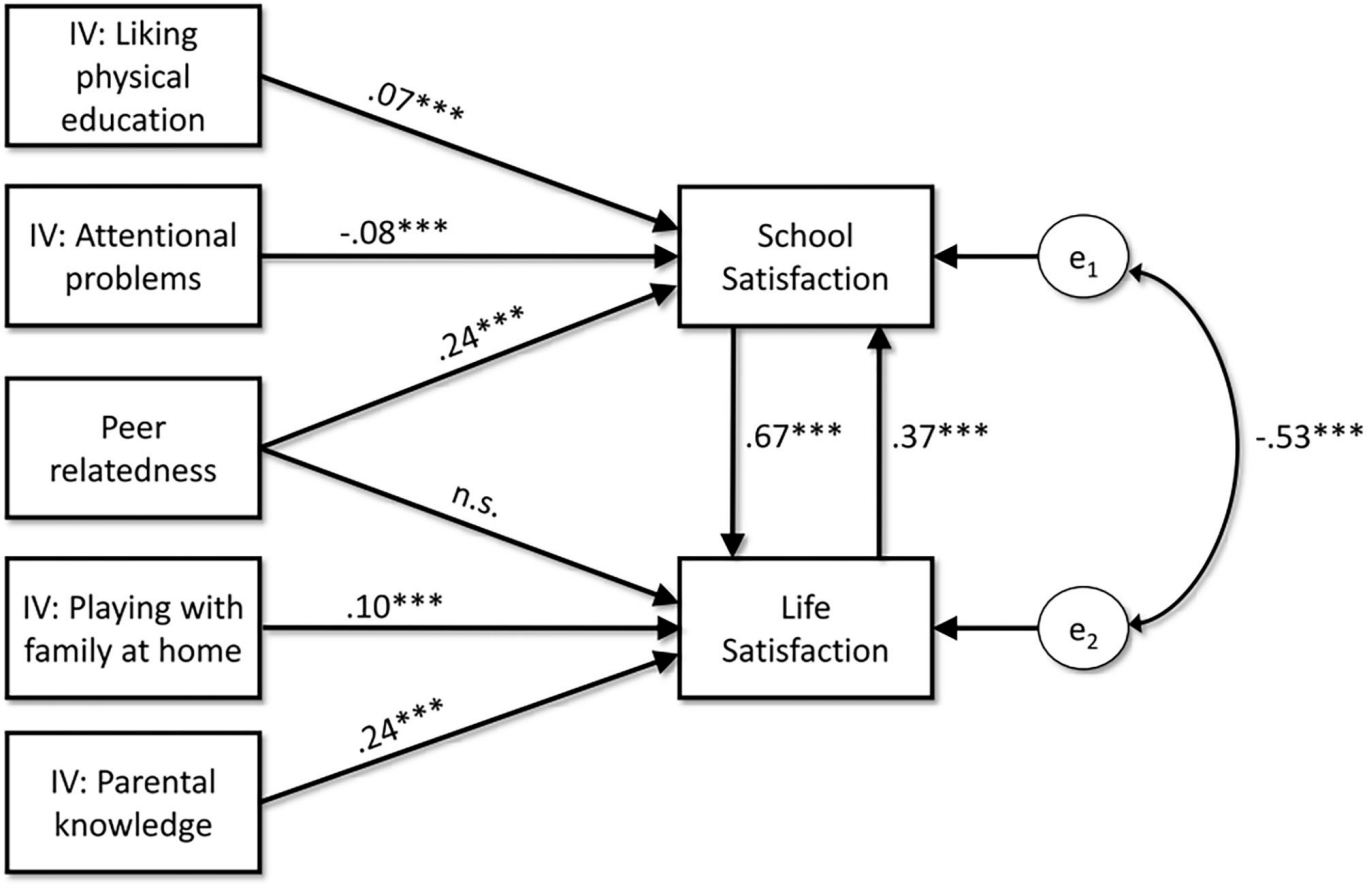
Path diagram of parameter estimates from SEM analysis. ***p < 0.001. The estimate indirect effect of peer relatedness on school satisfaction is β = 0.22 (p < 0.001).

Next, we estimate the model using 2SLS, 3SLS, and SEM methods, as previously described. The scaled Chi-Square, reveals that the absolute fit of the model is excellent [$\chi_{\text{S}-\text{B}}^{2}$ (2)= 0.01; *p* = 0.99] and, consequently, the relative fit indices are good as well (CFI = 1.00; TLI = 1.00; RMSEA = 0). Parameter estimates, standard errors, and *p*-values, shown in Table 2, are highly similar across SEM and econometric methods, suggesting a robust result. Because parameters similarity across methods, only SEM estimates are presented in Figure 2 and further discussed.

**Table 2:** Parameter estimates with econometric and SEM methodologies.

	**2SLS**			**3SLS**			**SEM**		
	**β**	***SE***	***p***	**β**	***SE***	***p***	**β**	***SE***	***p***
**Life satisfaction on**									
School satisfaction	0.67	0.09	<0.001	0.67	0.09	<0.001	0.67	0.11	<0.001
Peer relatedness	0.00	0.04	0.909	0.00	0.04	0.911	0.00	0.04	0.925
Parental knowledge	0.23	0.04	<0.001	0.24	0.04	<0.001	0.24	0.05	<0.001
Playing with family	0.10	0.03	<0.001	0.10	0.02	<0.001	0.10	0.03	<0.001
Intercept	−0.28	0.05	<0.001	−0.28	0.05	<0.001	−0.28	0.06	<0.001
*R*^2^	0.11			0.11			0.18^a^		
**School satisfaction on**									
Life satisfaction	0.37	0.10	<0.001	0.37	0.10	<0.001	0.37	0.12	0.002
Peer relatedness	0.24	0.03	<0.001	0.24	0.03	<0.001	0.24	0.03	<0.001
Attentional problems	−0.08	0.01	<0.001	−0.08	0.01	<0.001	−0.08	0.02	<0.001
Liking physical education	0.07	0.01	<0.001	0.07	0.01	<0.001	0.07	0.01	<0.001
Intercept	0.00	0.01	0.837	0.00	0.01	0.837	0.00	0.01	0.839
*R*^2^	0.25			0.25			0.25^a^		
Var (e.Life satisfaction)							0.88	0.07	
Var (e.School satisfaction)							0.75	0.03	
Cov (e.Life, School sat.)							−0.53	0.12	

*2SLS, Two Stage Least Squares; 3SLS, Three Stage Least Squares; SEM, Structural Equation Modeling; var, variance of error term; cov, covariance between error terms*.

a *Bentler-Raykov corrected R^2^*.

Regarding our first hypothesis (H1), the findings across the three methods confirm a significant reciprocal influence between school satisfaction and life satisfaction, with the impact from school to life satisfaction (β = 0.67*, p* < 0.001) being greater than the opposite effect (β = 0.37, *p* < 0.001).

Before moving on to the second hypothesis, we estimate a stability index (SI) to assess whether the variables do not influence each other successively in an infinite loop (Bentler and Freeman, 1983). The result (SI = 0.49) lies within the acceptable range [0, 1]; thus, we conclude that the model is in equilibrium and the mediational hypothesis (H2) can feasibly be analyzed.

As hypothesized, the results exhibited in Table 2 and Figure 2 confirm that peer relatedness has a significant direct effect on school satisfaction (β = 0.24, *p* < 0.001), but not on life satisfaction (β = 0.00, *p* = 0.93). Thus, the zero-order association between peer relatedness and life satisfaction (r =0.26, *p* < 0.001) can be fully explained by the mediational mechanism of school satisfaction, meaning that peer relatedness does not influence life satisfaction by itself but by first influencing satisfaction with school. This *indirect effect* is β = 0.22 (*p* < 0.001), meaning that in standardized scores, each unit of peer relatedness can explain around a fifth of standard deviation units of life satisfaction through the mediational effect of school satisfaction (see Paxton et al., 2011, for technical details about the estimation of indirect effects in non-recursive models).

We also tested the model's robustness to potential confounding effects of sociodemographic variables. For this purpose, we re-estimated the model, controlling the effect of age, gender, school grade, and type of school. The results, presented as Supplementary Material, shown that only gender (girls = 1) significantly affects life satisfaction (β = −0.13, *p* < 0.001) and school satisfaction (β = 0.09, *p* < 0.001), but without altering the pattern of effects exhibited in Table 2 and Figure 2. Thus, girls report lower life satisfaction but greater school satisfaction than boys, which is congruent with recent meta-analytic evidence (Chen et al., 2019).

## Discussion

Our findings support the premise of a reciprocal effect between school and life satisfaction. We are not aware of other studies demonstrating this reciprocal effect, but this line of inquiry deserves to be further addressed, as far is consistent with prior studies in adults, reporting reciprocal effects among domain and overall life satisfaction (e.g., Headey et al., 2005; Bowling et al., 2010; Chen et al., 2018; Bialowolski and Weziak-Bialowolska, 2020). If a reciprocal effect model is correct, using bottom-up or top-down models for analyzing the relationship between life satisfaction and domain satisfaction in children and adolescents is misguided.

From a theoretical perspective, Bronfenbrenner (1979; 2005) social-ecological approach helps contextualize the findings. The model proposes that individuals' development occurs within interconnected systems classified according to how close they are to their daily lives (micro-, meso-, exo- and macro-system). The micro-system includes the relationships an individual has direct contact with insides his/her immediate environments such as family and school. The meso-system encompasses the interactions among the structures of the micro-system. The social-ecological model emphasizes that interactions are bi-directional. Applied to the present research, the school can be conceived as a micro-system structure that directly influences early adolescents' evaluations of their life satisfaction. Those evaluations can then generate beliefs and expectations about other contexts, including the school, which influences school satisfaction evaluations.

From a practical point of view, our results imply that interventions aimed to improve school-related well-being can boost overall life satisfaction in early adolescents. Also, promoting good relationships among classmates and nurturing peer relatedness could influence not only early adolescents' satisfaction with their schools but also their subjective well-being. The findings can also be interpreted in a reversed way: broken social relationships in the school context and low peer relatedness can jeopardize school satisfaction and harm subjective well-being. On a policy level, the results suggest the importance of considering peer relatedness as a relevant intervention focus in public policies to improve children's and adolescents' quality of life.

It is worth considering some important limitations when interpreting the findings of this study. First, we acknowledge that this study's potential contribution is also a serious limitation: we assess reciprocal effects with cross-sectional data because only the third wave of ELPI includes questions on subjective well-being. Even if non-recursive models with cross-sectional data are useful tools, they have limitations and do not supersede using panel data and cross-lagged designs to test reciprocal causality.

A second limitation is the use of single-item scales to measure both overall life satisfaction and school satisfaction. Although single-item measures are popular in survey research, they have lower reliability and validity than multi-item scales. A related limitation is that our peer-relatedness measure was created *ad-hoc* from available items because the ELPI survey does not incorporate a validated peer relatedness scale.

Another limitation of this research is that we use data from only one country; thus, our results could be culturally specific and only describe Chilean early adolescents' school experiences.

Even given these limitations, this brief research report contributes to the literature on peer relatedness and the field of well-being by testing and confirming two hypotheses about the underlying mechanism through which peer relatedness can influence school satisfaction and life satisfaction in early adolescence. Furthermore, we illustrate the use of non-recursive models to test reciprocal effects with cross-sectional data using instrumental variables.

## Data Availability Statement

Data from Chilean Early-Childhood Longitudinal Survey (Encuesta Longitudinal de Primera Infancia, ELPI) 2017-2018 are publicly available upon request (http://www.elpi.cl).

## Ethics Statement

The ELPI survey was evaluated and approved by the Ministerio de Desarrollo Social y Familia, Chile, and by the Ethics Committee of the US National Institutes of Health (see http://www.elpi.cl for details). Written informed consent to participate in this study was provided by the participants' legal guardian/next of kin.

## Author Contributions

RG conceived the study, reviewed the relevant literature, developed the hypothesis, analyzed the data, interpreted the results, and wrote the manuscript's first draft. MG-C supervised every stage of the process. Both authors played an equal role in manuscript editing and made a substantial contribution to this manuscript.

## Conflict of Interest

The authors declare that the research was conducted in the absence of any commercial or financial relationships that could be construed as a potential conflict of interest.

## Footnotes

^1^We thank the reviewer that suggested these references.
