# Supplementary material for: Peer Relatedness, School Satisfaction, and Life Satisfaction in Early Adolescence: A Non-recursive Model
Source: Front Psychol. 2021 Mar 9;12:641714. doi: 10.3389/fpsyg.2021.641714 (PMC7985552; doi:10.3389/fpsyg.2021.641714)
Supplement: Supplementary file 1 [file Table_1.DOCX]

**Supplementary Tables**

Supplementary Table 1

*Means, standard deviations, and correlations of the variables used to develop peer relatedness scale*

| Items | Mean | SD | Min | Max | 1 | 2 | 3 | 4 |
| --- | --- | --- | --- | --- | --- | --- | --- | --- |
| 1. *How is your relationship with your classmates*? | 4.34 | 0.80 | 1 | 5 |  |  |  |  |
| 2. *My classmates think I have good ideas* | 0.81 | 0.39 | 0 | 1 | .26 |  |  |  |
| 3. *I feel lonely in the class* | 3.70 | 0.69 | 1 | 4 | .32 | .30 |  |  |
| 4. *I have a good time with my classmates* | 3.56 | 0.80 | 1 | 4 | .36 | .30 | .44 |  |
| 5. *I have a hard time in the classroom* | 3.63 | 0.71 | 1 | 4 | .31 | .26 | .47 | .37 |

Note. All correlations are significant with p < .001

Supplementary Table 2

*Factor loadings and Graded Response Model parameter estimates for peer relatedness scale*

| Items | λ | *a* | *b*_1_ | *b*_2_ | *b*_3_ | *b*_4_ |
| --- | --- | --- | --- | --- | --- | --- |
| *How is your relationship with your classmates*? | 0.61 | 1.30 | -4.54 | -3.8 | -1.64 | -0.07 |
| *My classmates think I have good ideas* | 0.59 | 1.25 | -1.46 |  |  |  |
| *I feel lonely in the class* | 0.84 | 2.65 | -2.26 | -1.78 | -1.00 |  |
| *I have a good time with my classmates* | 0.77 | 2.06 | -2.37 | -1.61 | -0.74 |  |
| *I have a hard time in the classroom* | 0.76 | 1.96 | -2.52 | -1.95 | -0.85 |  |

Note. λ = Factor loading; *a* = discrimination parameter; *b*_k_ = threshold parameters.

Supplementary Table 3

*Instrumental variables validity and strength*

|  | Sargan test | | Bassman test | | Stock & Yogo test | |
| --- | --- | --- | --- | --- | --- | --- |
| Instrumental variables | χ^2^ | *p-value* | χ^2^ | *p-value* | F | *p-value* |
| for *Life satisfaction* |  |  |  |  |  |  |
| Parental knowledge & Playing with family at home | 0.010448 | .918 | 0.010436 | .918 | 39.248 | < . 001 |
| for *School satisfaction* |  |  |  |  |  |  |
| Attentional problems & Liking physical education | 0.000031 | .995 | 0.000031 | .995 | 59.0563 | < . 001 |

Note. Sargan (1958) and Bassman (1960) are tests of overidentifying restrictions. The null hypothesis is that the instruments are uncorrelated with the error term of the predicted variable. Thus, failing to reject H0 supports the assumption of valid instruments. Stock & Yogo (2005) discuss a test of IVs' predictive strength whose null hypothesis is that the instruments are weak. As a rule of thumb, F values above 10 suggest strong IVs. From Stock & Yogo (2005) critical values, an F statistic greater than 19.93 is recommended to guarantee the IVs' strength. Altogether, current results suggest that IVs are valid and strong.

**References**

Basmann, R. L. (1960). On Finite Sample Distributions on Generalized Classical Linear Identifiability Test Statistics. *Journal of the American Statistical Association, 55*, 650–59.

Sargan, J. D. (1958). The estimation of economic relationships using instrumental variables. *Econometrica: Journal of the Econometric Society*, 393-415.

Stock, J. H., & Yogo, M. (2005). Testing for weak instruments in linear IV regression. In J, H. Stock & D. W. K. Andrews (Eds.), *Identification and Inference for Econometric Models: Essays in Honor of Thomas Rothenberg*. (pp. 80-108). Cambridge, UK: Cambridge University Press.

Supplementary Table 4

*Parameter estimates with SEM methodology, including age as covariate*

|  | β | *SE* | *p* |
| --- | --- | --- | --- |
| *Life satisfaction on* |  |  |  |
| School satisfaction | .66 | .11 | <.001 |
| Peer relatedness | .00 | .04 | .911 |
| Age | -.01 | .02 | .658 |
| Parental knowledge | .24 | .05 | <.001 |
| Playing with family | .10 | .03 | <.001 |
| Intercept | -.18 | .23 | .413 |
|  |  |  |  |
| R^2^ | .18 |  |  |
|  |  |  |  |
| *School satisfaction on* |  |  |  |
| Life satisfaction | .36 | .12 | <.001 |
| Peer relatedness | .24 | .03 | <.001 |
| Age | -.01 | .02 | .688 |
| Attentional problems | -.08 | .02 | <.001 |
| Liking physical education | .07 | .01 | <.001 |
| Intercept | .08 | .20 | .699 |
|  |  |  |  |
| R^2^ | .25 |  |  |
|  |  |  |  |
| var(e.Life satisfaction) | .88 | .07 |  |
| var(e.School satisfaction) | .75 | .03 |  |
| cov(e.Life, School sat.) | -.53 | .12 |  |

Note. χ^2^_S-B_ = 0.01; *p* = .99

Supplementary Table 5

*Parameter estimates with SEM methodology, including gender as covariate*

|  | β | *SE* | *p* |
| --- | --- | --- | --- |
| *Life satisfaction on* |  |  |  |
| School satisfaction | .68 | .11 | <.001 |
| Peer relatedness | .00 | .04 | .999 |
| Gender (1 = girls) | -.13 | .03 | <.001 |
| Parental knowledge | .25 | .05 | <.001 |
| Playing with family | .10 | .03 | <.001 |
| Intercept | -.23 | .06 | .413 |
|  |  |  |  |
| R^2^ | .18 |  |  |
|  |  |  |  |
| *School satisfaction on* |  |  |  |
| Life satisfaction | .35 | .12 | .003 |
| Peer relatedness | .25 | .03 | <.001 |
| Gender (1 = girls) | .09 | .03 | .001 |
| Attentional problems | -.08 | .02 | <.001 |
| Liking physical education | .07 | .01 | <.001 |
| Intercept | -.05 | .02 | .008 |
|  |  |  |  |
| R^2^ | .25 |  |  |
|  |  |  |  |
| var(e.Life satisfaction) | .88 | .07 |  |
| var(e.School satisfaction) | .75 | .03 |  |
| cov(e.Life, School sat.) | -.52 | .12 |  |

Note. χ^2^_S-B_ = 0.55; *p* = .76

Supplementary Table 6

*Parameter estimates with SEM methodology, including school grade as covariate*

|  | β | *SE* | *p* |
| --- | --- | --- | --- |
| *Life satisfaction on* |  |  |  |
| School satisfaction | .67 | .11 | <.001 |
| Peer relatedness | .00 | .04 | .932 |
| School grade | .00 | .01 | .774 |
| Parental knowledge | .23 | .05 | <.001 |
| Playing with family | .10 | .03 | <.001 |
| Intercept | -.30 | .10 | .002 |
|  |  |  |  |
| R^2^ | .18 |  |  |
|  |  |  |  |
| *School satisfaction on* |  |  |  |
| Life satisfaction | .37 | .12 | .002 |
| Peer relatedness | .24 | .03 | <.001 |
| School grade | .00 | .01 | .845 |
| Attentional problems | -.08 | .02 | <.001 |
| Liking physical education | .07 | .01 | <.001 |
| Intercept | -.02 | .07 | .819 |
|  |  |  |  |
| R^2^ | .25 |  |  |
|  |  |  |  |
| var(e.Life satisfaction) | .88 | .07 |  |
| var(e.School satisfaction) | .75 | .03 |  |
| cov(e.Life, School sat.) | -.52 | .12 |  |

Note. χ^2^_S-B_ = 0.01; *p* = .99

Supplementary Table 7

*Parameter estimates with SEM methodology, including school type as covariate*

|  | β | *SE* | *p* |
| --- | --- | --- | --- |
| *Life satisfaction on* |  |  |  |
| School satisfaction | .64 | .11 | <.001 |
| Peer relatedness | .01 | .04 | .725 |
| School type (1 = public) | -.01 | .03 | .762 |
| Parental knowledge | .25 | .05 | <.001 |
| Playing with family | .11 | .03 | <.001 |
| Intercept | -.29 | .06 | <.001 |
|  |  |  |  |
| R^2^ | .18 |  |  |
|  |  |  |  |
| *School satisfaction on* |  |  |  |
| Life satisfaction | .33 | .12 | .006 |
| Peer relatedness | .25 | .03 | <.001 |
| School type (1 = public) | .01 | .02 | .547 |
| Attentional problems | -.09 | .02 | <.001 |
| Liking physical education | .07 | .01 | <.001 |
| Intercept | -.01 | .02 | .552 |
|  |  |  |  |
| R^2^ | .25 |  |  |
|  |  |  |  |
| var(e.Life satisfaction) | .88 | .07 |  |
| var(e.School satisfaction) | .75 | .03 |  |
| cov(e.Life, School sat.) | -.52 | .12 |  |

Note. χ^2^_S-B_ = 0.01; *p* = .99
